# Histopathologic Aspects of Malignancy-Associated Granuloma Annulare: A Single Institution Experience

**DOI:** 10.3390/dermatopathology10010015

**Published:** 2023-03-04

**Authors:** Buket Bagci, Cansu Karakas, Harsimran Kaur, Bruce R. Smoller

**Affiliations:** 1 Department of Pathology and Laboratory Medicine, Loma Linda University, Loma Linda, CA 92354, USA; 2 Department of Pathology and Laboratory Medicine, University of Rochester Medical, Rochester, NY 14642, USA

**Keywords:** granuloma annulare, paraneoplastic granuloma annulare, paraneoplastic, malignancy-associated granuloma annulare, granulomatous inflammation, malignancy associated

## Abstract

Granuloma annulare (GA) is a benign, self-limiting granulomatous inflammatory disease that exhibits different histologic patterns. Infrequently, granuloma annulare can be associated with malignancy, the so-called malignancy-associated granuloma annulare (MGA). In this study, we aimed to compare the clinical and histopathological differences between GA and MGA. We retrospectively reviewed patient charts and identified 35 patients diagnosed with GA and concurrent hematological or solid organ malignancies as a case group. Additionally, we identified 33 patients without any known solid organ or hematological malignancy as a control group. MGA is commonly seen in the seventh decade of life, while GA affects the younger population. MGA is most commonly presented in the extremities of the body. The most common malignancy associated with MGA was chronic lymphocytic leukemia. Prostate cancer was the most common type of solid organ malignancy that was associated with MGA. The most common histopathological pattern seen in MGA was interstitial, comprising half of the cases. Multinucleated giant cells were present in half of the MGA cases and in most of the control group. In the literature, there are no established features that distinguish MGA from GA. Although MGA and GA have overlapping features, in our series, we found that the interstitial pattern was more common in MGA, while the necrobiotic pattern was more common in GA.

## 1. Introduction

Granuloma annulare (GA) is a common, benign, self-limiting entity. It was first described by Colcott-Fox in 1895, and the term “granuloma annulare” was used by Radcliffe-Crocker in 1902. Women are more likely to be affected than men (F/M ratio: 2/1) [1]. In 1980, dermatologists reported that 0.1% to 0.4% of their new patients presented with GA [2]. Furthermore, this study is based on patients presenting to dermatology clinics. Therefore, it may underestimate the prevalence and incidence, and large population-based studies to estimate the true prevalence and incidence are still lacking. Although it can present at any age, it occurs more commonly in children and young adults. The hands, arms, feet, and legs are the most commonly affected locations [2,3,4]. Localized, generalized, and subcutaneous clinical types are the most common; perforating, acral, patch, and follicular pustule forms present less often [1,4,5,6].

**Figure 1:**
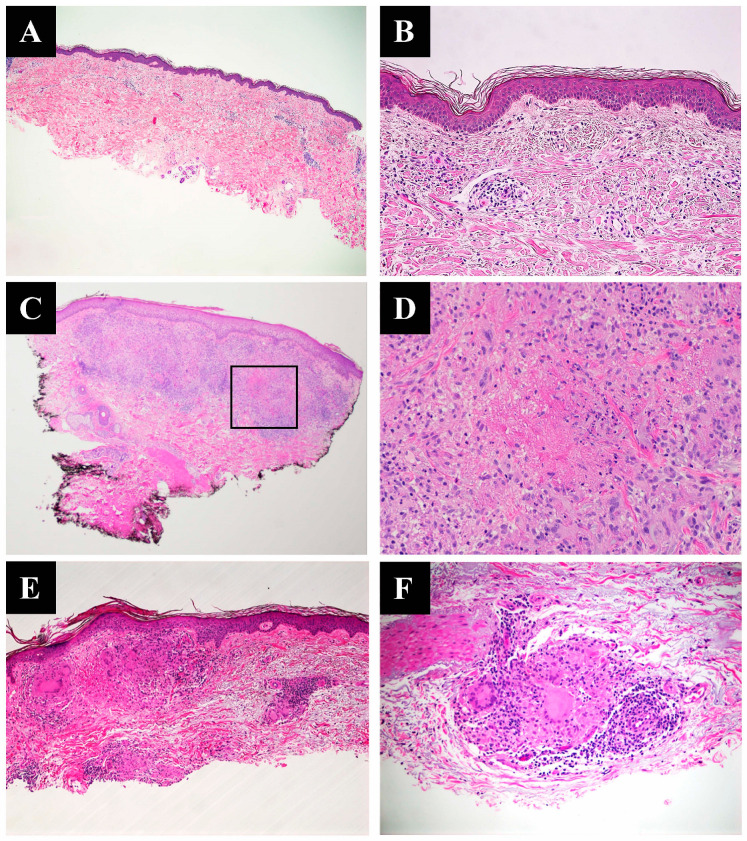
(**A**) A shave biopsy demonstrates spindled and epithelioid histiocytes interspersed between degenerated collagen bundles, extending from the superficial dermis to the lower reticular dermis (H&E, 4×). (**B**) High-power image of interstitial GA (H&E, 20×). (**C**) Lower- power image of a punch biopsy shows necrobiotic granulomatous inflammatory infiltrate (H&E, 2×). (**D**) Foci of eosinophilic necrobiotic collagen surrounded by a peripheral histiocytic palisade and giant cells (H&E, 20×). (**E**) A shave biopsy demonstrates well-formed sarcoidal granulomas composed of islands of epithelioid cells associated with multinucleated giant cells from the superficial to the reticular dermis (H&E 4×). (**F**) Higher-magnification image of a sarcoidal granuloma with perivascular inflammation (H&E, 20×).

Histologic variants include necrobiotic (collagenolytic) GA, interstitial GA, and sarcoidal GA, also known as tuberculoid GA. Histologically, interstitial GA exhibits an interstitial histiocytic infiltrate with the degeneration of collagen, increased connective-tissue mucin intercalating between collagen bundles, and a lymphocytic infiltrate (Figure 1A,B). Necrobiotic GA exhibits palisading granulomas with central necrobiosis and may present with increased connective tissue mucin; this is the classical and most easily identified pattern (Figure 1C,D). Sarcoidal GA exhibits aggregated epithelioid histiocytes with a sharply punched-out granulomatous appearance (Figure 1E,F).

The pathogenesis of this disease remains unknown; however, delayed-type hypersensitive reactions and cell-mediated reactions have been implicated in the literature [7,8]. GA is associated with infectious diseases, metabolic diseases and disturbances, autoimmune diseases, and hematologic and solid organ malignancies [6]. The first reported case of MGA was associated with Hodgkin’s lymphoma. Associations of MGA with other malignancies have also been reported. Although some case reports and a few small studies have addressed this issue, histopathological differences between GA and MGA are still not clear. In this study, we report a large and diverse set of MGA cases and explore the clinical and histopathological differences between GA and MGA.

## 2. Materials and Methods

This study was approved by the RWJ Barnabas Health and the University of Rochester Institutional Review Board. To identify cases, we retrospectively reviewed patient charts to determine clinical information and pathology reports for the period between 1 January 2006 and 31 October 2020. We selected all patients previously diagnosed with GA by the dermatopathology service. We identified a subset of cases in which GA was identified at any time after a visceral or hematologic malignancy diagnosis. In two cases, GA was diagnosed 3 and 5 months before the diagnosis of malignancy. Radiologically, however, there was evidence of a neoplasm that was later discovered to be breast carcinoma and renal cell carcinoma. We excluded patients under 18 years of age. Thirty-nine biopsies from thirty-five patients met these criteria and were included. Additionally, we identified GA cases without known visceral or hematologic malignancy as the control group. Thirty-six biopsies from thirty-three patients were identified and included in the control group for comparison. All these cases were re-reviewed by a board-certified dermatopathologist (BRS) to confirm the diagnosis. We created a template to identify the following: location and type, density of perivascular inflammatory infiltrate, the presence of mucin deposition, the presence of multinucleated giant cells and eosinophils, and associated malignancies.

We categorized histopathologic types as either necrobiotic, interstitial, sarcoidal (tuberculoid), or mixed. We used the “mixed” classification for biopsies that revealed more than one of the clearly defined subtypes. We categorized “mild perivascular inflammatory infiltrate” as those cases exhibiting few lymphocytes in the region surrounding the vessels in the superficial vascular plexus. We categorized “moderate perivascular inflammatory infiltrate” as those cases where the lymphocytic infiltrate was apparent at low magnification but restricted to a perivascular distribution. We categorized “intense perivascular inflammatory infiltrate” as those cases where a brisk infiltrate extended beyond the vessels into the interstitial collagen. We used the two-tailed *t*-test for continuous clinical data and chi-square test for categorical data. We compared the outcomes of the case and control groups. Features with a *p*-value < 0.05 were considered significant.

## 3. Results

### 3.1. Case–Control Study

We evaluated 39 biopsies from the MGA group and 36 biopsies from the control group. Table 1, Table 2 and Table 3 present our clinical and histopathological findings of the case group and the control group.

### 3.2. Clinical Characteristics

The mean age of the patients in the case group was 72 years (59–90 years old), and the gender distribution was close to even (F:18, M:21). The mean age in the control group was slightly younger, 63 years (18–93 years old) (t(73) = 2.8, *p*-value < 0.0056). There was a statistically significant female predilection (F:25, M:11) (t(73) = 2, *p*-value < 0.04). The upper extremity was the most commonly affected location (17/39), followed by the trunk (12/39) and the lower extremities (9/39) (Table 1). In one case, the location was not disclosed. The most commonly affected body locations of the control group were the lower extremities (14/36), followed by the upper extremities (12/36) and the trunk (10/36) (Table 1).

Four patients had more than one malignancy at the time of diagnosis, while thirty-one patients had only one malignancy. Nineteen cases were associated with hematological malignancy, including eight chronic lymphocytic leukemia, three acute myeloid leukemia, three myelodysplastic syndrome myeloproliferative disease, three cutaneous T-cell lymphoma, one chronic myelomonocytic leukemia, and one B-cell lymphoma. Twenty-three cases were associated with solid organ malignancies. Of these, seven (18%) were prostate carcinomas, five (13%) were breast carcinomas, five were cutaneous melanoma, three (8%) were genitourinary carcinomas, two (5%) were gynecologic carcinomas, and one (3%) was lung carcinoma (Table 2).

### 3.3. Histopathological Characteristics

**Figure 2:**
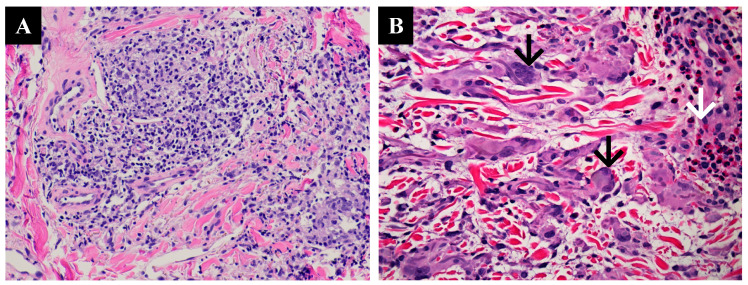
(**A**) A sarcoidal granuloma with severe perivascular inflammation (H&E, 20×). (**B**) A skin biopsy shows multinucleated giant cells (black arrows) with eosinophilic infiltrate (white arrow).

As stated above, we categorized the MGA cases and controls into histopathologic types. In the MGA cases, we found seventeen cases of interstitial GA (17/39, 44%), fifteen cases of necrobiotic GA (15/39, 38%), three cases of sarcoidal/tuberculoid GA (3/39, 8%), and four cases of mixed GA (4/39, 10%) (Table 2). The statistical analysis did not show any significance. Fifteen cases exhibited mild perivascular inflammatory infiltrate, nineteen cases exhibited moderate perivascular inflammatory infiltrate, and five cases exhibited intense perivascular inflammatory infiltrate (Figure 2A). Multinucleated giant cells were present in eighteen patients (46%) (Figure 2B). Mucin was observed in twenty-nine patients (74%). Eosinophils were present in only three cases. A dermatomyofibroma adjacent to the MGA was observed in one case. In another patient, we noted perifollicular abscesses with deep muscular involvement. In this case, the granulomatous nature of the GA was at some distance from the follicular abscesses, and we judged this to be an unrelated condition. Finally, we observed perineural lymphocytic inflammation in a single patient.

In the control group, we found eighteen biopsies were necrobiotic GA (50%), fourteen were interstitial GA (39%), three were mixed GA (8%), and one was sarcoidal GA (3%). All of the controls showed some degree of perivascular inflammatory infiltrate (mild 11/36. moderate 15/36, and intense; 10/36). Thirty-three cases of the control group showed mucin. Mucin is more likely to be seen in GA (*p*-value: 0.047). Twenty-eight cases of the control group showed multinucleated giant cells, and multinucleated giant cells are more likely to be present in idiopathic GA (*p*-value: 0.22).

## 4. Discussion

To the best of our knowledge, this is the largest investigative study of MGA to date. Idiopathic GA and MGA most commonly affect the extremities of the body [6,9]. A study of idiopathic GA by Wells and Smith showed that the condition was most commonly exhibited in the upper extremities (60%) and the lower extremities (20%) of the body. On the contrary, in our series, lower extremities were more likely to be involved in the control group and upper extremities were more likely to be involved in the MGA cases. The average age for patients with generalized GA is 37 with bimodal presentation in the first and fifth decades of life [10]. The largest previous study of idiopathic GA found the greatest incidence and prevalence was in the fifth decade of life [11]. Our findings were consistent with previous studies, with MGA presenting in an older age group (mean age: 72 years old), while GA presented in a younger population (mean age: 63 years old). Additionally, GA shows a female predominance, while MGA shows no differences in terms of gender. Multiple studies and case reports have demonstrated a prominent predilection for MGA in elderly patients, mostly in the seventh decade of life [12,13]. Most malignancies are also more common in elderly patients. Almost 80% of all malignancies are diagnosed in patients over the age of 55, and so, the presence of GA might be considered coincidental [14]. However, MGA demonstrates a resistance to treatment with conventional therapy and yet resolves with the treatment of malignancy, which suggests a relationship between malignancy and GA [13,15]. Finally, no reports in the literature link GA to malignancies in younger population groups.

The pathogenesis of GA is not well understood. Some theories have been proposed to explain GA development. One such hypothesis is that cell-mediated reactions cause T-helper cells to differentiate into cells that express TNF-α and matrix metalloproteinases and to release interferon, which activates macrophages and causes tumor necrosis factor stimulation [7,16,17]. This theory is supported by the finding that GA can be ameliorated by downregulating the helper T cell-1 cytokine pattern with TNF inhibitors [16]. Another suggestion is that the presence of immunoglobulins and their complement, which cause blood vessel wall necrosis, fibrinoid change, and nuclear dust, are related to immune complex deposition and may be involved in pathogenesis. This hypothesis would account for the finding of perivascular inflammatory infiltrate in virtually all GA cases [18]. Further support for this proposed mechanism has been provided by the observation of GA development following checkpoint inhibitor administration [17].

GA is associated with non-neoplastic and neoplastic conditions. Non-neoplastic conditions include chronic diseases such as diabetes mellitus, hyperlipidemia, thyroid disease, and infections [6]. In terms of neoplastic conditions, GA may coexist with hematological or solid organ malignancies. Hodgkin’s lymphoma was first reported in association with GA, and subsequent reports later suggested associations with a range of different hematological malignancies [12,19,20]. Researchers have also reported associations with various solid organ malignancies such as hepatocellular, lung, breast, and prostate carcinoma and gastrointestinal stromal tumor [21,22,23,24,25,26,27,28,29]. In our study, the most common type of malignancy associated with GA was chronic lymphocytic leukemia (20%), and the most common visceral malignancy was prostate cancer (18%). These findings do not conflict meaningfully with the literature. Other solid organ malignancies associated with GA included endometrial, breast, ovarian, renal, and urothelial carcinomas. In contrast to previous studies, in which lung cancer was the most common visceral malignancy associated with MGA, we found only one case. As the numbers in our series were not large, this finding may have more to do with the demographic features of our population than any statistically significant association [21,22,23,24,25,26,27,28,29]. We reviewed all MGA cases from the National Library of Medicine Database in English without any time limitations. We identified 75 cases of GA associated with malignancy. The most common malignancy associated with MGA was lymphoma (48%), followed by myeloid hematological malignancy (14%), lung cancer (12%), breast cancer (10%), neural malignancy (5%), melanoma (5%), gastrointestinal malignancy (4%), gynecological malignancy (2%), and testicular cancer (1%). These figures correlate reasonably close with the findings of our study.

When we carried out a histopathologic examination of the MGA group, we found almost half of the cases demonstrated an interstitial GA pattern, and the second most commonly seen pattern was necrobiotic GA (39%). In cases of solid tumors, interstitial GA (31%) was slightly more prevalent than necrobiotic GA (26%), while hematolymphoid malignancies exhibited no differences. Cases associated with melanoma tend to present a necrobiotic pattern. However, the number of melanoma cases was very small, and these results might not be representative. Although our findings did not reveal any statistical significance, Mangold et al. concluded that the interstitial type (70%) was more common in MGA cases, while the necrobiotic type (60%) was more common in idiopathic GA cases [13]. These findings may be related to a small number of cases. Additionally, a Korean study of 54 cases found that generalized GA is more likely to present as necrobiotic GA (52%) than interstitial GA (48%) [10]. Consistent with previous studies, our control group exhibited a predominance of necrobiotic GA (14/36). On the contrary, Winkelmann et al. studied 207 GA cases and found that 71% of GA cases were interstitial and 26% were necrobiotic GA [1]. Additionally, Winkelmann et al. found varying degrees of perivascular mononuclear inflammatory cell infiltration in all 207 of their GA cases [1]. We also found varying degrees of perivascular inflammatory infiltrate in all of the MGA and control group biopsies. Mangold et al. found an association between perivascular inflammatory infiltrate and MGA [13]. They found perivascular inflammatory infiltrate was present in 70% of the MGA group but only in 15% of the control group [13]. Nonetheless, they considered perivascular inflammatory infiltrate to be a rare finding in MGA [1]. Although perivascular inflammatory infiltrate is a prominent feature in MGA, idiopathic GA may also exhibit perivascular inflammatory infiltrate; therefore, perivascular inflammatory infiltrate does not help to distinguish MGA from GA. Mucin was present in most of the MGA and control groups (74% and 91%, respectively). Another study of generalized GA found mucin was present in 94% of cases and eosinophils in 44% [10]. We found eosinophils were a rare feature in both the MGA group (3/39) and the control group (1/36).

Multinucleated giant cells were seen in approximately half of the MGA cases (46%) and in most of the control cases (77%). Mangold et al. found only one in seven MGA cases showed the presence of multinucleated giant cells. In contrast to these findings, the review of 207 cases by Winkelmann et al. found only a few GA cases with multinucleated giant cells [1]. However, the difference in multinucleated giant cells might be explained by the small number of MGA and GA cases in our series. The formation of multinucleated giant cells can be observed in cases of neoplasia, inflammation, and some infections. Monocyte fusion has been found to be the cause of multinucleated giant cell formation, which is led by interferon-γ [30]. Interferon-γ plays a significant role in the immune defense mechanism of neoplasia [31]. The tumor microenvironment that is caused by interferon-γ might explain why multinucleated giant cells were present in MGA cases but absent in most idiopathic GA cases in prior studies. However, the relatively small size of our study population means that this increased presence of multinucleated giant cells in the control or MGA group may not be significant. More extensive studies are required before any unequivocal conclusions can be stated.

## 5. Conclusions

In summary, our data showed that MGA is more prevalent in the seventh decade of life, while GA presents in the younger population. We found no differences in terms of location. There is a diversity in the histopathologic patterns. Interestingly, idiopathic GA is more likely to exhibit a necrobiotic GA pattern, while MGA shows no particular pattern. One of the unique findings in our study is that idiopathic GA is more likely to exhibit mucin and multinucleated giant cells than MGA. Additional large studies are warranted to further elucidate the distinctive histopathologic manifestations of this entity.

## Figures and Tables

**Table 1:** Clinicopathologic findings of the MGA cases.

Clinicopathologic Findings		Interstitial GA (n:17)	Necrobiotic GA (n:15)	Sarcoidal GA (n:3)	Mixed GA (n:4)	Overall (n:39)
**Mean AGE**		74 (y)	70 (y)	70 (y)	73 (y)	72 (y)
**Location**	Upper extremities	7 (18%)	7 (18%)	1 (3%)	2 (%)	17 (%)
	Lower extremities	4 (10%)	3 (8%)	2 (5%)	0 (0%)	9 (23%)
	Trunk	6 (15%)	5 (13%)	0 (0%)	1 (3%)	12 (31%)
**Perivascular Inflammation**	Mild	10 (26%)	4 (10%)	0 (0%)	1 (3%)	15 (38%)
	Moderate	4 (10%)	9 (23%)	3 (8%)	3 (8%)	19 (49%)
	Severe	3 (8%)	2 (5%)	0 (0%)	0 (0%)	5 (13%)
**Multinucleated Giant Cells**		4 (10%)	10 (26%)	2 (5%)	2 (5%)	18 (46%)

**Table 2:** Histopathologic patterns of the MGA cases and associated malignancy types.

Type of Malignancy		Interstitial GA (n:17)	Necrobiotic GA (n:15)	Sarcoidal GA (n:3)	Mixed GA (n:5)	All (n:39)
**Hematologic Malignancies**	CLL	2	5	0	1	8(20%)
	AML	2	1	0	0	3 (8%)
	MDS/MPN	2	1	0	0	3 (8%)
	CMML	1	0	0	0	1 (3%)
	MF	0	0	1	2	3 (8%)
	B cell lymphoma	0	0	1	0	1 (3%)
	Overall	7	7	2	3	
**Solid Organ Malignancies**	Breast carcinoma	4	0	1	0	5 (13%)
	Prostate carcinoma	3	3	0	1	7 (18%)
	Ovarian carcinoma	0	1	0	0	1 (3%)
	RCC	0	1	0	1	2 (5%)
	Urothelial	1	0	0	0	1 (3%)
	Melanoma	2	3	0	0	5 (13%)
	Lung carcinoma	1	0	0	0	1 (3%)
	Endometrial carcinoma	1	0	0	0	1 (3%)
	Overall	12	8	1	2	

CLL—chronic lymphocytic leukemia; AML—acute myeloid leukemia; MDS/MPN—myelodysplastic syndrome- myeloproliferative neoplasm; CMML—chronic monomyelocytic leukemia; MF—mycosis fungoides; RCC—renal cell carcinoma.

**Table 3:** Clinicopathologic findings of the control group.

Clinicopathologic Findings		Interstitial GA (n:14)	Necrobiotic GA (n:18)	Sarcoidal GA (n:1)	Mixed GA (n:3)	Overall (n: 36)
**Mean AGE**						63 (yo)
**Location**	Upper extremities	3 (%)	7 (%)	0 (0%)	2 (%)	12 (%)
	Lower extremities	9 (%)	4 (%)	1 (%)	0 (0%)	14 (%)
	Trunk	2 (%)	7 (%)	0 (0%)	1 (%)	10 (%)
**Perivascular Inflammation**	Mild	3 (%)	7 (%)	1 0%)	0 (%)	11 (%)
	Moderate	6 (%)	9 (%)	0 (0%)	0 (%)	15 (%)
	Severe	5 (%)	2 (%)	0 (0%)	3 (%)	10 (%)
**Multinucleated Giant Cells**		8 (22%)	14 (39%)	1(3%)	3 (8%)	26 (77%)

## Data Availability

The data presented in this study is available on request from the corresponding author.
